# Trait Philosophique de Medecine Pratique

**Published:** 1841-01-01

**Authors:** 


### Traité Philosophique de Medecine Pratique. Par *A. N. Gendrin,* D.M. Medecin de l'Hôpital de la Pitié. Tomes 2, Bailliere, Paris, 1839.

" Philosophical Treatise "—We are so little accustomed on this side of the Channel with authors styling themselves and their works *philosophes* and *philosophiques,* that we are always amused with the quiet and gentlemanly assurance of our lively neighbours in this respect.

The practice of late years has been so common with them, that the terms, we suppose, are really not intended to imply much more than the usual " most obedient," or " most humble," at the close of a business letter. We meet with Traités Philosophiques in great abundance; M. *Bouillaud* recently published a Philosophie Medicale; there are not a few works on Anatomie Philosophique, and Physiologie Philosophique; and soon, doubtless, we shall have philosophical treatises on surgery and midwifery, and ultimately perhaps on separate diseases, so that ere long we may be called upon to notice some philosophical work on uterine hæmorrhage or stone in the bladder!

As far as we know, this foolish practice has prevailed chiefly among the medical authors of late years in France; the literary men seem to be aware of the universal distrust and dislike into which their *philosophe* predecessors of the revolutionary æra have fallen, to have any wish to resume the appellation.

That there are works in medical literature, to which the title of philosophical might be justly applied, we do not deny; but their authors had too much of the modesty of genuine science, to claim it for themselves or for their productions. Need we allude to the immortal writings of Hunter, Bichat, and Laennec? How far the present treatise of M. *Gendrin* may be considered by himself worthy of being associated with these, we cannot tell; but, without much prophetical presumption, we think that we may assure him that *when* they are forgotten, but not till then, his will have a fair chance of enduring renown.

This "Traité Philosophique" is announced to consist of four volumes; two only have as yet appeared. "It is to embrace and systematise all the parts of medicine which are specially applicable to the knowledge and treatment of diseases." After a few introductory observations, a great portion of which is far too recondite for us to understand, the author expounds his classification of diseases; dividing them into two great divisions—the first comprehending those maladies which consist in an alteration of the functions of Organic life; and the second, those which consist in the alteration of the functions of Animal life. These two divisions embrace nine classes. 1. Hæmorrhagiæ—2. Diacrises or alterations of Secretion—3. Phlegmasiæ—4. Pyrexiæ, or Fevers—5. Anomalotrophies, or alterations of nutrition—6. Heterosarcoses, or formations of Accidental Tissues—7. Cachexiæ—8. Neuroses—and, 9. Vesaniæ, or Mental Disorders. The first seven appertain to the functions of organic life, and the two last to those of animal life.

This nosological arrangement is in our opinion one of the most faulty which we have ever met with, and is liable to innumerable objections; as in one place it associates diseases which have little in common with each other, and, in another, it disjoins those which are practically and essentially very much alike. Most readers will be surprised to observe that the pyrexiæ are postponed to the hæmorrhagiæ, and to the phlegmasiæ—rather a strange fancy; seeing that fever is a necessary accompaniment of all the phlegmasiæ, and also of all active hæmorrhages. But we cannot stop to canvas the merits and demerits of M. *Gendrin's* system; we need only state that the whole of the first volume before us and more than one-half of the second are occupied with the description of the hæmorrhagiæ alone; commencing with epistaxis, and successively treating of hæmoptysis, gastro-entero-hæmorrhagia, hæmaturia, hæmorrhoids, hæmorrhage from the skin, then apoplexy, pneumo-hæmorrhagia, dysmenorrhœa, menorrhagia, and, lastly, uterine hæmorrhage during and after pregnancy.

Had our limits permitted, we intended to have made some remarks on the chapter upon the general description of hæmorrhage. It is very imperfect; especially as respects the most practical part of the subject, the treatment.

But indeed this might have been looked for; as the same defect may be predicated of almost every work on practical medicine which issues from the French press. It is truly astonishing how far behind their brethren, not only in England but also in Germany, the French physicians seem to be on every branch of therapeutics. For example, M. *Gendrin* seems scarcely to be aware of the utility of nauseants in arresting hæmorrhage; although they are unquestionably among the most powerful means that can be employed.

We believe that many physicians are deterred from the administration of these medicines by the fear of vomiting being induced, and of the hæmorrhage being thereby aggravated. This fear, we may assure them, is in almost every case groundless; we have repeadly administered emetics of ipecacuan and the tartate of antimony in almost every form of hæmorrhage, including hæmoptysis, hæmatemesis, and menorrhagia, not only without danger, but generally with the most beneficial results. Vomiting has in general the effect not of increasing, but of decidedly arresting, discharges of blood. The nausea that precedes it has a most powerfully sedative effect on the actions of the heart, and on the whole circulation; and even the violent efforts of the stomach and respiratory muscles during the act are not observed to accelerate the force of the arterial circulation, but only to retard for a short time the return of the blood along the veins. The nausea should always be kept up for many hours subsequently, by the use of repeated small doses of the antimony.

Another important omission of M. *Gendrin* is that he does not even notice such remedies as the acetate of lead, or the sulphate of zinc or of alum in the treatment of hæmorrhages: he seems to think that the internal use of such remedies is almost entirely nugatory. In this, he is much mistaken. That the effect of these salts, when properly administered, is in many cases most speedily to arrest discharges of blood, cannot be disputed by any one who has fairly tried them. For example, we have repeatedly seen hæmaturia, chronic as a matter of course, checked by the use of alum in the course of a day or two; and many cases of menorrhagia, which had continued long, have very rapidly been cured by the administration of sulphate of zinc in the form of pills, associated with dilute sulphuric acid and the tincture of hyosciamus.* But we must stop in these strictures; and we shall now direct the attention of our readers to one really valuable chapter, at the commencement of M. *Gendrin's* second volume, on the *Physiology of Menstruation.* His observations on this topic are minute and satisfactory; and the conclusions which he draws from them—although they cannot be regarded as strictly original—seem to be quite legitimate, and withal are very interesting.

---

* These results cannot appear surprising, when we remember that many chemical salts are so rapidly absorbed into the system, and may be detected in the urine and other secretions within a very short time after being swallowed. The recent researches of M. *Orfila* on Poisons, wherein he has proved that the salts of arsenic, antimony and copper, may be detected, not only in the blood and urine, but even in the substance of the viscera and muscles, lead to many important deductions in practical therapeutics.

The most important of these conclusions is that *in every instance of genuine menstruation there is a rupture of one of the Graafian vesicles on the surface of the ovary,* and that this rupture is accompanied with a dilatation of the corresponding Fallopian tube, an approximation of its loose end to the ovary, the presence of a red-coloured mucus within the tube and the uterus, and the development of villosities, probably of a vascular nature, on the inner surface of the latter organ. According to this view, the catamenial discharge is not the primary or essential act in the curious function of menstruation: it is merely the evidence and effect of a change that has already taken place in one of the ovaries.

The reason, therefore, on the one hand, that menstruation does not occur before the age of puberty is, that up till this period no distinct vesicles are observed in the ovaria; and, on the other hand, that it ceases at what is called the critical age in women, is that these organs then become atrophied and otherwise altered in structure.

The following are the data observed by M. *Gendrin,* and from which these interesting conclusions have been drawn.

A woman, 30 years of age, committed suicide by hanging herself, on the 8th of February, 1828, while the catamenia were upon her.

On dissection, the mucous surface of the vagina and cervix uteri was found highly injected; the uterus contained a sanguinolent mucus, and its internal surface, especially at the fundus, exhibited numerous fungiform villosities of a reddish grey colour, which were best seen under water. The right ovarium and Fallopian tube presented nothing unusual; but the left tube was found to be considerably dilated and to contain a reddish coloured mucus, which was most abundant at its open extremity. The ovary too on this side was observed to be highly injected at one point of its surface to the extent of a quarter of an inch; and, in the centre of this vascular spot, there was a distinct fissure or cleft of about a line and a half across, and provided with loose or fringed edges. This fissure led into a small cavity, which might receive a hemp-seed, and whose parietes were of a bright red colour: it was evidently a Graafian vesicle which had become ruptured. Four other vesicles, unbroken, and each of about the size of a hemp-seed, were found in the substance of the ovary.

In a *second case,* which was that of a girl 19 years of age, who died suddenly while menstruating, the appearances in the vagina and uterus were exactly similar to what we have noticed in the preceding case. "The Fallopian tubes were filled with a reddish mucus; the *morsus diaboli* of the right one was applied to its ovary, the surface of which exhibited a highly injected network of bloodvessels, and at one point a minute solution of continuity leading into a small cavity of about two lines in diameter. The vascular network on the surface of the ovary was most distinct and of the deepest colour for two or three lines around the fissure. Three vesicles, two as large as hempseeds and the other of the size of a pin's-head, were found in the substance of this ovary; the larger ones being nearest to its surface, and the smaller one most deep-seated. In the left ovary were observed, at varying depths, five vesicles, each of about the size of a millet-seed: no trace of any cicatrix on its surface was visible. The reddish mucus contained in the canal and the *morsus* of the Fallopian tube was examined with a powerful magnifying-glass; but nothing like an organised body could be detected."

In a *third* case, which occurred in a woman, 27 years of age, who met with her death from a severe accident on the fourth day of menstruation, we are informed that "the right ovary exhibited two distinct but incomplete (inachevées) cicatriculæ—one depressed and umbilicated, and which presented the vestige of a minute central excavation; the other of a yellow hue, and underneath it there was an empty cavity or *loge* of a line and a half in diameter, and whose walls were of a yellowish-red colour. Two or three injected capillaries were seen on the surface of the ovary between these cicatriculæ. Five entire vesicles were found in the left, and only one in the right ovary."

In the *fourth* case, a girl 20 years of age died from pneumonia on the third day after the appearance of the catamenia, which had remained only twenty-four hours upon her.

The cavity of the uterus was occupied with semi-coagulated blood, but only colourless mucus was found in the Fallopian tubes. The right ovary exhibited a minute fissure, beneath which there was a dilated locular cavity of about two lines in diameter, whose edges were red and *tomentose* when examined under water: a narrow vascular areola surrounded the fissure. Only one Graafian vesicle was found in this ovary. The left one, which was unusually small, exhibited no traces either of a fissure or of any vesicles.

The *fifth* and last case, reported by M. *Gendrin*, is that of a woman, 44 years of age, and mother of three children, who died from apoplexy on the second day after the appearance of the catamenia, which had been always quite regular, and lasted on most occasions for three days.

"The uterus and vagina contained semi-coagulated blood; numerous vascular villosities were seen on the internal surface of the former. The left tube was dilated and full of reddish mucus; its *morsus* was applied to the ovary, on the surface of which was observed a fissure leading into a minute excavation which was filled with a similar fluid. This *locule*, examined under water, seemed to be about a line in depth; it formed the centre of a vascular areola on the surface of the ovary of four or five lines in diameter. Three vesicles were formed in the substance of this ovary. The other one exhibited no appearance of any fissure, and only two vesicles were found in it."

M. *Gendrin* informs us that he has made numerous examinations of the ovaries in young girls before the age of puberty, and that on no occasion has he ever found any appearances similar to those which we have now described. In three cases only has he observed any vesicles: the girls were above 12 years of age, but had not menstruated, and presented none of the outward appearances of puberty. In these three cases the ovaries, only very partially developed, were found to contain from one to four minute vesicles of the size of small pins'-heads, and which were deeply imbedded in their substance.

M. *Gendrin* next describes the progressive changes in the structure of the Fallopian tubes and ovaries in women after the cessation of the catamenia, remarking that, " anatomists are agreed as to the absence of the Graafian vesicles in those who have passed the critical period of life;" and he sums up the results of his examinations of the ovaries in menstruating women in the following words:—

" Dissection has repeatedly verified in them the presence of vesicles in the ovaries, (the larger ones being always nearest to the surface,) and the existence of cicatriculæ on their surface in different states of development—from the red vascular cicatriculæ, exhibiting an irregular central depression, to that which is evident only from having a yellowish hue and a slight loss of smoothness and polish of the ovarian surface. Whenever the women have had their catamenia at from one to several weeks before death, I have uniformly found that the cicatricula was most recent and most vascular in those who had most recently menstruated.

In some women, whose catamenia have been interrupted for several months, no vesicles are found in the ovaries, and we cannot discover any trace of cicatriculæ: generally, however, under such circumstances, we observe very small and deeply-seated vesicles. Occasionally we observe, imbedded in the substance of the ovaries, cells filled with a brownish-yellow sanies-like matter, resembling a small coagulum partially softened and discoloured."

The inference drawn by our author from the preceding observations is, that the function of menstruation is essentially and necessarily connected with, nay rather dependent upon, the development, maturation, and rupture of the peculiar vesicles formed in the ovaries,* and that the immediate cause of the secretion is the plethoric condition of the uterus thereby induced, and the manifestation of vascular villosities on its inner surface.

*John Hunter* had long ago remarked the *hyperæmic* state of the uterus, and the tendency to exudation of blood on its surface, in women who had died during menstruation; but it was *Joerg* who first described in these cases the vascular-like villosities to which we have so frequently alluded. But neither of these distinguished writers seems to have been aware that the phenomena were necessarily associated with the rupture of an ovarian vesicle and the destruction of an ovum arrived at maturity.

Such is a summary of the very interesting observations of M. *Gendrin* on menstruation. It is but due, however, to other authors to observe that these observations are not entirely novel and original. One English writer in particular, and he of no mean note on every subject of uterine pathology —Dr. *Robert Lee*—has unquestionably preceded him, at least in point of publication.

It is indeed rather strange that no reference should be made by M. *Gendrin* to the valuable paper by Dr. *Lee* in the Cyclopædia of Medicine, as we have a right to expect that the translator of two of our standard works, *Thomson* on Inflammation, and *Abercrombie* on Diseases of the Brain, should be tolerably well acquainted with British medical literature. But the ignorance on the part of French writers of any literature save their own is so prevailing, that we are unwilling to suspect M. *Gendrin* of any want of good faith on the present occasion, and we attribute his omissions rather to neg-

* The appearance of cicatriculæ on the surface of the ovaries must therefore, according to this view, be regarded only as a sign of menstruation, and not necessarily of conception, having ever taken place, as has been so long imagined.

lect than to dishonesty. Unquenchable vanity is the besetting sin of the French people; it pervades their every action, whether in war and politics, or in the more peaceful domains of literature and science. Nowhere is it more conspicuous than in the writings of their medical men. At almost every meeting of the Academy of Medicine in Paris, we find that controversies are carried on upon various topics, which to the learned members seem to have all the interest of novelty, but which are known to every pupil of every respectable school in this country. For example, there was a most elaborate discussion last year on the treatment of fever with purgatives, and many of the members talked of the importance of this discovery!—seemingly not at all aware of Dr. *Hamilton's* admirable work published nearly half a century ago. It may therefore be unfair to suppose that M. *Gendrin* was aware of Dr. *Lee's* paper, and that the omission of any reference to it was wilful; but surely the author of a "Traité Philosophique" should make himself acquainted with the works of the more distinguished writers of former and present times. Waving however the question as to priority of discovery, we may remark that the circumstance of more than one writer unaware probably at the time of each other's researches, having come to the same conclusions as to the physiology of menstruation, must be regarded as a strong argument in favour of their accuracy. As the subject is one not only of great curiosity but of practical importance, we shall devote a page or two to a brief exposition of the observations contained in Dr. *Lee's* interesting paper.

After mentioning several circumstances which very clearly point out the intimate connexion between the function of menstruation and the condition of the ovaries—as for example the non-appearance of the catamenia in women in whom the ovaria have been found on dissection to be wanting, and their utter cessation in the remarkable case where Mr. Pott extirpated the organ in an operation for hernia; also the regular recurrence of periodic pains through the pelvis, similar in every respect to those which often attend menstruation, in certain cases where the uterus has been afterwards found to be either entirely absent or only imperfectly developed—and alluding to the curious changes which are known to occur in hen birds in which the ovaria have become shrivelled from disease, he proceeds to detail some very interesting observations which he has made on the anatomical appearances of the ovaria in women, who have died while the catamenia were upon them. The *first* seem to have been made in March 1831,* in the case of a young woman who died, while menstruating, from phlebitis.

.......... "The left ovarium was larger than the right, and at one point a small circular opening with thin irregular edges was observed in its peritoneal coat, which led to a cavity of no great depth in the ovarium. Around the opening, to the extent of three or four lines, the surface of the ovarium was of a bright red colour, and considerably elevated above the surrounding part of the peritoneal coat. On cutting into the ovarium, its substance around the opening and depression was vascular, and several *Graafian* vesicles of different sizes were observed. The right ovarium was in the ordinary state. Both Fallopian tubes

---

* It is but fair to M. *Gendrin* to remind the reader that the first of his observations is dated in the year 1828: the dates of his other cases are not given.

were intensely red and swollen and their cavities were filled with menstrual fluid. The lining membrane of the uterus was coated with the same fluid, and the parietes were soft and vascular."

Nothing can be more satisfactory than these details; and while they anticipate in the date of publication, they beautifully confirm the observations of M. *Gendrin*. There is one circumstance in the case now mentioned that deserves notice; the right Fallopian tube contained menstrual fluid, although there was no appearance of rupture on the surface of its ovary. Was this owing to a consentaneous action of the two tubes? However this may be, we find that again, in Dr. *Lee's second* case, "the free extremities of the Fallopian tubes were gorged with blood, and their cavities were filled with a red-coloured fluid," although the right ovary only exhibited the appearance of a recent fissure or laceration on its surface. In a subsequent case, the details of which are minutely given by the author, and where the opening was found in the left ovary, menstrual fluid was present in the left tube only, although "both tubes were red and gorged."

The inference of M. *Gendrin* that the development and maturation, so to speak, of the Graafian vesicles is in proportion to their proximity to the peritoneal coat of the ovary, *seems* to be confirmed by the following observation of Dr. *Lee* in the case of a woman who died from cholera, while menstruating.

"The ovarium," he says, "was much larger than natural, and at one point there was a small irregular aperture in its peritoneal coat, through which a portion of a slender coagulum of blood was suspended. On cutting into the substance of the ovarium, it was found to be occupied by three small cavities or cysts, one of which was filled with a clear ropy fluid, another with semifluid blood, and the third, which communicated with the opening in the peritoneal coat, with a firm coagulum."

Dr. *Lee* closes his interesting observations with the following wisely-cautious words:—

"The facts which have now been related render it extremely probable that all the phenomena of menstruation depend upon, or are connected with, some peculiar changes in the Graafian vesicles, in consequence of which an opening is formed in their peritoneal and proper coats. Whether an entire vesicle, or only the fluid it contains, escapes through this opening at the period of menstruation, further observations may hereafter determine."*

He appends a useful practical hint:—

"In many cases of disordered menstruation, chlorosis, and hysteria which we have observed, the symptoms have been clearly referable to certain morbid

---

* Dr. *Lee*, it should be mentioned, does not claim to himself the originality of these views. He most candidly admits that, as far back as the year 1797, Mr. Cruikshanks has distinctly recorded that, in the case of a young woman who died with the menses upon her—"the external membranes of the ovary were burst at one place, from whence I suspect an ovum escaped, descended through the tube to the uterus, and was washed off by the menstrual blood." In reference to the latter part of this statement Dr. *Lee* remarks, that "there is no proof whatever that an ovum passes along the Fallopian tube into the uterus during menstruation, and it is not clearly established that this takes place even subsequent to conception."

states of the uterine appendages, and decided benefit has resulted from the application of those local remedies which were employed with the view of subduing the irritation, congestion, or inflammation which appeared to be present in those parts of the uterine system."

From the extracts which we have given from Dr. *Lee's* paper, the reader must see how perfectly his observations coincide with those of M. *Gendrin.* Their accuracy is further confirmed by the researches of Dr. *Negrier* of Angers, who has recently published a small work, entitled "Recherches Anatomiques et Physiologiques sur les Ovaires dans l'Espece Humaine, considerés specialement sous le rapport de leur Influence dans la Menstruation, p. 130, avec 11 planches lithographiées." Professor *Negrier* claims for himself the priority of the discovery, as he has been in the habit, he tells us, of describing in his lectures for the last nine or ten years the dependence of the catamenial secretion upon certain changes in the ovaries. We are not however aware that he ever published his views till last year. The following is a summary of his work, for which we are indebted to the pages of the Gazette Medicale.

The ovarian vesicles in mammiferous animals were usually regarded as representing the ovum in the greater number of animals of other classes, until the period when MM. *Prevost* and *Dumas* discovered, in two cases, the ovulum in the interior of the vesicle. The former opinion was not however wholly abandoned till after the researches of *Baer,* who pointed out the precise spot where the ovulum is observed within the vesicle, and described it very exactly. The subsequent labours of *Coste,* and also of *Valentin* and *Bernhard,* have carried us a step further in our knowledge, by determining the situation of the proligerous vesicle at one point of the ovulum, where it is so extremely minute that it does not exceed 376 ten-thousandth parts (376 dix milliemes) of a line in dimensions.

According to the opinion hitherto generally entertained, the Graafian vesicles are slowly developed in the substance of the ovary, until the moment of conception, when one of them gives exit to an ovulum, which is then conveyed along the Fallopian tube into the cavity of the uterus: the cicatrix, thereby left at the point of rupture, assuming a yellowish appearance, and well known under the name of *corpus lutcum.* Great attention has been paid by many pathologists to the characteristic features of these yellow spots, which when distinctly marked have usually been regarded as a certain proof that the woman has at some time or another conceived. The most satisfactory description of the genuine *corpora lutea* will be found in Dr. *Montgomery's* excellent work on the Signs of Pregnancy, and in Dr. *Robert Lee's* valuable paper in the 22nd vol. of the Medico-Chirurgical Transactions.

In the first chapter of his work, Dr. *Negrier* describes the gradual and successive development of the ovaria from the earliest periods of life up to puberty; and in the second, the changes which these organs undergo at the first appearance of the catamenia, and during the whole period of fecundity.

From the facts narrated in this latter chapter, it would seem that, at certain epochs, an afflux of transparent fluid takes place into the cavity of the most superficial vesicle; this becomes in consequence distended, and at length gives way at the point where the investing parietes of the ovarium are thinnest and most yielding. The ruptured point is usually quite cicatrised, at least outwardly on the peritoneal surface of the ovary, in the course of from eight to ten days. The rupture of the vesicule is followed by a slight effusion of blood in its interior from the minute vessels, which have given way. This seems to be very quickly absorbed; as the little cavity is sometimes found empty, and communicating with the peritoneum. The cicatrix subsequently assumes a yellow appearance, which remains for some time. Now these appearances, Dr. *Negrier* says, are never observed when the catamenial secretion is suspended, as for example, during pregnancy and lactation, or when it has completely ceased, after what has been called the critical epoch of life in women. The conclusion which he draws from his researches is, that the evolution and rupture of the Graafian vesicles is the cause of menstruation, and that all the symptoms of this important function are attributable to the changes that are successively going on in the ovaries, and to the sympathetic irritation in the uterus which is thereby induced. After alluding to the circumstance that, if the uterus of a woman during the act of menstruation be examined, we always observe that the sanguineous congestion of its substance is uniformly more decided on the side corresponding with that ovary in which one of the Graafian vesicles is ruptured, and especially around the opening of the Fallopian tube of that side, Dr. *Negrier* remarks, " From an extended series of observations, I feel confident in stating, as an indubitable fact, that *in the ovaries of women who have menstruated, of whatever age they may be, vesicular cicatrices never fail to be found.* The catamenial secretion is so completely dependent on the functions of the ovary that, if this organ is not duly developed, it is always retarded; and, if on dissection we find the abortive formation of some vesicles, we may be certain that the returns of the catamenia have been of late irregular and interrupted. Again, if we examine the ovaries of girls, in whom the appearance of the secretion has been precocious, we shall find them more than usually developed, and in every respect like what we observe in women of a marriageable period of life. On the contrary, in those in whom menstruation is tardy and difficult, the ovaries are found to be small, and their parenchyma to exhibit only traces of vesicular evolutions imperfectly developed." At the critical period of life, it is rare that the catamenial secretion ceases all at once; usually its returns are for a period irregular, and the quantity of the discharge varies much, being at one time very scanty, and at another time very profuse. Now the examination of the ovaries at this period of life exhibits corresponding irregularities in the development of the Graafian vesicles: some have evidently aborted before their maturity, and have either remained in the state of grey pouches or cells, (bourses grises) or look like yellow vesicles.

We may conclude from this circumstance that the incomplete development has not been sufficient to induce a full re-action in the uterus; and hence is the imperfection and irregularity of the secretion. However, if some of the vesicles attain a complete maturity, the development is tardily effected, and may thus induce that state of plethoric engorgement which causes those profuse hæmorrhages, so frequent towards the close of the menstrual function. One of the cases reported by Dr. *Negrier* seems to afford strong presumption of the truth of his opinions.

It was that of an unmarried woman, 50 years of age, in whom the hymen was quite entire, and in whom the catamenia had ceased for about three years before death: the ovaries were found on dissection of the size of almonds, and their surfaces were entirely covered with cicatrices, like deep grooves or furrows; one of them exhibited a small nucleus of the colour and hardness of baked earth.

Having thus occupied so much of the present article with these recently published opinions and observations on the physiology of menstruation, we cannot now spare above a page or two to the notice of some of the other contents of M. *Gendrin's* volumes, which otherwise might have detained us longer.

We have already said that the whole of the first volume, and more than one-half of the second are occupied with a description of the hæmorrhagiæ. We select what the author says of the least common form of hæmorrhagic disease—*Hæmatidrosis*, or exudation of blood from the skin. The chapter on this subject is more complete than any we have met with elsewhere: and, as the disease is seldom treated of in systematic works, we shall give our readers a short account of the most interesting cases collected together by M. *Gendrin.*

*Case* 1.—A girl, eleven years of age, was seized with a sharp pain in the right arm, which soon became covered with numerous pustules. A few days afterwards blood was observed to ooze from their surface; and then they all disappeared without leaving any marks. The next month the same symptoms returned, and these were quickly followed by the first appearance of the catamenia. The following month, there was a similar return of these phenomena, and in the same succession. For some time afterwards there was no re-appearance of the hæmorrhage from the arm; but during the following winter, whenever the fingers of the right hand became very cold, blood oozed from their tips, although there was not the slightest trace of any crack or wound in the skin. By merely warming the hand, the oozing was stopped, and, as spring approached, this curious tendency to hæmorrhage ceased altogether. For four months the catamenia returned regularly; but then they ceased. Again the oozing of blood from the right fingers returned, sometimes every or every second day, and at other times only every eight days. It was impossible to detect any orifice from which it flowed. Some time after this date, the girl was seized with vertigo, flushing of the face, tumefaction of the neck, &c. These symptoms were succeeded and relieved by an oozing of blood from several points on the front of the neck. At another time, epistaxis came on, which did not cease until again the neck began to swell, and the oozing of blood from its surface to return. On the same day a bloody fluid exuded from the skin of the right arm, and from the calf of the left leg. At this period the catamenia had been absent for several months, and the girl was subject to a variety of sufferings, such as cramps, partial paralysis, &c.

On one occasion, the left eye became amaurotic, and the tears from this eye were sanguinolent. On another occasion, the skin of the nose exuded blood, and this was followed first by epistaxis, and then by hæmoptysis. Subsequently there was an oozing from under the nails of the right hand, and from the skin of the right arm: and this, some time afterwards, was followed by a trifling hæmorrhage from the left eye, and the skin of the right hand.*

A curious case is related by Dr. *Boivin* in the Dictionnaire des Sciences Med. t. iv.

A middle-aged woman, after a blow on the stomach, was attacked with hæmatemesis. For fifteen years, the hæmorrhage returned at shorter, or longer intervals. At length it ceased, after the administration of some powerful astringent medicines. But now ensued an exudation of blood from various parts of the surface of the body and limbs;—from the front of the chest, the back, the thighs, legs, feet, and toes. The catamenia at this period were quite regular in their return. While this cutaneous exudation continued the woman felt well; but she was immediately affected with various distressing feelings when it ceased. A pruritus in the part usually preceded the oozing from the skin. The woman was 48 years of age when Dr. *Boivin* saw her, and it was then nearly three years since the first appearance of this singular cutaneous discharge. The catamenia had ceased for about two years, but the cessation of these had not affected the recurrence of the Hæmatidrosis. It was chiefly from the scalp, the upper part of the chin, and from about the angles of the jaw, that the oozing took place, when this gentleman wrote. Twice during the day the patient felt a sense of heat and an itchiness in these parts; the skin became then somewhat swollen, and blood oozed from its pores in large drops. The health of the patient seemed entirely good.

In the same work is narrated the case of a middle-aged man, whose constitution had been much enfeebled by intense study and mental distress, and in whom, after a sexual debauch, the thighs, axillæ, pubis, and particularly the penis itself, became excessively painful. From all these parts, especially from the glans, blood oozed out in a stream. This hæmorrhage returned every fortnight or three weeks for the next 20 months, and usually lasted for two or three days at a time. The issue of the case is not given.

The next case occurred in a man 28 years of age. One evening he had drunk wine to excess, and had remained all the following night in a state of intoxication: he had vomited several times. Next morning, after a violent fit of rage, he felt a dull pain, accompanied with pruritus, on the left side of the chest near the armpit. On applying his hand to the part, he was surprised to find it covered with blood. He went immediately to the Hôpital de la Pitié, and we (M. *Gendrin ?*) found that the blood was flowing in drops from the surface of the left armpit and the adjoining part of the chest. When wiped clean, the skin was observed to be slightly swollen and red. The drops of blood formed slowly; they were almost confluent, and soon joined together. This cutaneous hæmorrhage continued the whole day; so that the quantity of blood lost amounted, it was believed, to three pounds. There was no fulness of the pulse, nor any disturbance of the general health. Next day, the hæmorrhage had ceased, and the man seemed quite well.

Occasionally this form of hæmorrhage, Hæmatidrosis, is observed in young

* *Van Swieten,* Comment. in *Boerhaave,* Aphor. t. iv.

infants. The following case is reported by M. *Eggerdes.* An infant, three weeks old, had become very much emaciated, when one morning the sleeve of its shirt was found spotted with blood, although no trace of any wound or injury could be seen. The child seemed better, and sucked with more strength than it had done before. Next day, the right arm was found covered with blood. This sanguineous exhalation continued for the following five or six days; and each day the child's health seemed to be improved. The left arm then became the seat of a similar hæmorrhage, which continued for a few days: ultimately the little patient recovered perfectly.

As illustrative of the occasional causes of sanguineous exsudation, we may mention that *Leroux* alludes to the case of a man, employed at the porcelain manufactory at Sevres, whose perspiration became quite sanguinolent whenever he exposed himself to the heat of the furnace. Violent muscular exercise will sometimes produce the same effects. *Latour* tell us of a man in whom fencing brought on an exsudation of blood from the surface of the body; and in the Ephemerides Nat. Cur. we read the case of a young girl, who, after excessive dancing, was similarly affected. Strong mental impressions have been known to act in the same way. (The record by the Evangelist of the Agony in the Garden, that " He sweat, as it were, great drops of blood," may have been literally true.) Excessive pain also will give rise to this curious disease. Thus Dr. *Caizergues* has related the instance of a woman, in whom, while suffering under severe nephritis, drops of blood oozed from the skin of the face and of various parts of the body. The most frequent cause however of Hæmatidrosis is certainly suppression of the catamenia. In the majority of cases, the hæmorrhage has been more or less distinctly periodic, and has gradually ceased, when the menstrual function was restored to a healthy state. The disease does not require any specific course of treatment. Its cause should, as a matter of course, be first ascertained, and, by removing it and bringing the general system into a healthy state, the cutaneous hæmorrhage will gradually subside, and ultimately disappear.

From the extracts which we have given from M. *Gendrin's* work, it will be perceived that it is by no means destitute of valuable information. As a whole, it does not please us; in parts, it is excellent.

